# Milk-supply from the Bacteriological Standpoint

**Published:** 1898-04

**Authors:** M. P. Ravenel

**Affiliations:** Bacteriologist, State Livestock Sanitary Board of Pennsylvania; Instructor in Bacteriology in Veterinary Department, University of Pennsylvania


THE JOURNAL

OF

COMPARATIVE MEDICINE AND

VETERINARY ARCHIVES.

VOL. XIX. *APRIL, 1898.* No. 4.

### MILK-SUPPLY FROM THE BACTERIOLOGICAL STANDPOINT.

By M. P. Ravenel, M.D.,

BACTERIOLOGIST, STATE LIVESTOCK SANITARY BOARD OF PENNSYLVANIA; INSTRUCTOR IN BACTERIOLOGY IN VETERINARY DEPARTMENT, UNIVERSITY OF PENNSYLVANIA.

The problem of a pure milk-supply for the people in general, and more particularly for our infant population and invalids, is unquestionably one of the most important matters of sanitation which confronts the hygienist of to-day. The enormous quantity which is used in all civilized countries attests its value as a food-medium, and if this supply is contaminated by disease-producing germs its influence for harm is enormous. In Great Britain, it is said, 250,000,000 gallons are consumed each year, while in the United States 5,209,125,567 gallons are used, an amount nearly equal to the Croton water used in the City of New York each month.

Until about the year 1870 milk was not generally considered as being capable of carrying epidemic disease, though as early as 1859 an outbreak of typhoid fever was reported, but since that time the evidence on this point is overwhelming as well as appalling. Mr. Ernest Hart gives statistics of fifty epidemics of typhoid fever, with 3500 cases; of fifteen epidemics of scarlet fever, with 800 cases; seven epidemics of diphtheria, with 500 cases. Taking up the work where Mr. Hart left off, Dr. Freeman, of New York, has collected statistics of fifty-three epidemics of typhoid fever, with 3226 cases; twenty-six of scarlet fever, with 1593 cases; eleven of diphtheria, with 501 cases; two epidemics of foot-and-mouth disease, three of throat-trouble, two of acute poisoning, and one of Asiatic cholera—in all about 736 cases. These

[1] Read at the annual meeting of the Pennsylvania State Veterinary Medical Association, March, 1898.

figures are inside of the real truth, as in many of the epidemics reported the number of cases occurring is not given.

It would be comparatively easy to outline ideal conditions for a milk-supply; but the commercial aspect of the case must also be considered, and the regulations enforced must be of a nature which will allow so important a food-product to be within the reach of the masses. An understanding of the principles on which an ideal supply could be produced, and of the dangers to which milk is exposed, will enable us to practise precautions which must tend to the betterment of the traffic as at present conducted. In giving warning of the dangers which may be incurred by the use of milk, I would not be understood as in any way opposing the use of milk as a food. On the contrary, I consider it the most perfect single food which we have, and strongly advocate its general use as a food-supply. I point out the dangers to better enable us to guard against them and to make this most valuable food still more desirable.

The proper place to begin the care of milk is unquestionably the animal that produces it, and some of the greatest dangers with to which we are exposed from the use of milk originate with the animal itself. Cows as food-producers should have most careful attention as regards their food, housing, and general surroundings. They should be under the constant supervision of a competent veterinarian, who should keep strict guard on their general health. The cows should have at least as much care as is bestowed on our horses, and yet as compared with horses the cows are usually very much neglected. The food often given is not fit even for pigs, and through the use of such refuse material bad flavors and smells not infrequently pass into the secretion, and cases of sickness have been reported on good authority from the use of such milk, although, as a rule, the senses only are offended. Many drugs also pass into the milk, and every one is aware of the garlicky odor given to the milk of cows feeding upon the wild onion.

More important, however, are the diseases to which the cow is subject, the germs of which may pass into the milk, through the secreting organ, and thus be conveyed to the consumer. Chief among these is tuberculosis, and it has recently been stated by Prof. James Long that probably more deaths occur each year from the drinking of tuberculous milk than would be caused by England's going to war with a first-class power. Without going into arguments to prove his position, I may state briefly the facts which lead up to such a conclusion. First, the tubercle bacillus has been repeatedly shown in the milk of cows having general tuberculosis, but without perceptible disease of the udder. Second, that both by inoculation and also by ingestion of such milk tuberculosis can be produced in several of the lower animals. Third, that the disease as seen in animals and as seen in man is essentially the same and has the same origin. Fourth, that in the large majority of children dying of tuberculosis the initial lesion is in some part of the digestive tract, pointing to food as a medium of infection. Children being large consumers of milk, it is hard to escape the conclusion that it is to them a frequent source of infection.

Two other diseases may also be mentioned. Klein has shown that the diphtheria bacillus may also pass into the milk through the udder of the cow, and although his work has not been confirmed, it has never been successfully refuted. The milk of cows suffering from some inflammatory diseases of the udder has also been shown to be the cause of disease in young persons similar to, if not identical with, scarlet fever. Such an instance was the epidemic occurring at Marylebone, St. Pancras, and Hampstead, which was traced to the milk of a farm at Hendon. On examination a micrococcus was found which seemed to be the cause of the trouble.

This list, by no means complete, will serve to show the importance of guarding well the health of milch-cows. As secreted normally by a healthy cow, milk is free from bacterial life; but even in the udder it is exposed to contamination from outside influences, and from the moment of drawing, and even during drawing, it is constantly exposed to bacterial contamination of various sorts. The majority of the epidemics before mentioned have occurred by contamination of the milk during the period between the drawing and delivery to the consumer, and it is in regard to this that reforms are especially needed. Milk is a most excellent culture-medium for almost all germs, but I doubt if the rapidity of multiplication of germs is realized by most people. Freudenreich has found as many as 577,500,000 germs in a single cubic centimetre of milk kept at 25° C. for twenty-four hours, and it is not unusual to find from 33,000,000 to 56,000,000 per pint in milk as ordinarily sold. It is not a pleasant thought, yet it is nevertheless true, that milk as it reaches the consumer is usually richer in bacteria than the sewage of our great cities. For instance, Sedgwick and Batchelder report fifty-seven samples of Boston milk which contained from 30,000 to 4,220,000 per cubic centimetre. Loveland and Watson, in the milk of Middletown, Conn., found from 11,000 to 85,500,000 per cubic centimetre, while the milk of Madison, Wis., ranges from 15,000 to 2,000,000 per cubic centimetre. Sedgwick found that the sewage of Lawrence, Mass., contained from 100,000 to 4,000,-000 per cubic centimetre. Bitter puts down 50,000 germs per cubic centimetre as the maximum limit for milk fit for food, while the committee in charge of the production of "certified milk" in Buffalo sets the limit at 10,000 per cubic centimetre. The majority of these are, of course, in themselves harmless, but many of them bring about the changes in milk which render it unfit for use and injure its keeping qualities.

The chief sources of these germs are the animal itself, the hands of the milker, and the dust of the stable. Contamination from these three sources can be easily very much lessened by attention to the simplest precautions of cleanliness. The hairy coat of the animal offers exceptional opportunities for the collection of dust and dirt, which often contain feces of the animal. This becomes attached to them when lying down, and is exceptionally rich in germ-life. These germs may be almost entirely gotten rid of by keeping the udder of the cow, inside the legs, and the belly clipped; before each milking the parts should be well brushed and moistened with a wet sponge, the excess of water being wiped off. Bacteria do not rise from moist surfaces, and the moisture serves to hold in place those germs which have escaped the brushing. In an experiment tried by Prof. Russell, of the University of Wisconsin, it was found that where a cow was milked without any special precautions, 5250 germs per minute were deposited upon an area equal to the exposed top of a ten-inch milkpail. Where precautions similar to the above were taken only 115 germs per minute were deposited upon the same area. In a plate exposed at a short distance from the cow, as a control experiment, only sixty-five germs per minute were deposited.

The hands of the milker and his clothing are also frequent sources of contamination. The hands should be carefully washed with soap and water, and a special garment, such as a blouse, or what is known as the ordinary "linen duster," should be put on just before milking. These should, of course, be frequently laundered.

The fore-milk has also great effect on the germ-contents of the entire milk. From the conformation of the teat, the milk-duct is open to the atmosphere, so that particles of filth may almost directly carry their germ-life to the milk-duct. Here they have suitable culture-media, and the warmth of the animal to facilitate growth, and though the number of species is generally small the fore-milk is usually rich in bacterial life. In one instance Prof. Russell found in the fore-milk 2800 germs per cubic centimetre, while the average of the total yield was only 330. The lesson is obvious; the fore-milk should be discarded, and especially to be deprecated is the too common habit of using the fore-milk to lubricate the hands and teat of the cow to facilitate milking. If a lubricant is needed a small amount of vaseline should be used, which has the additional advantage of retaining any germs with which it may come in contact.

For the prevention of contamination through dust in the stable, it would be better to have a separate milking-room kept exclusively for that purpose; but where this is not possible, much can be done by keeping the stable clean, and by avoiding the stirring up of any dust through the feeding of hay, cornstalks, or dry food of any sort just before milking. As soon as the milk is drawn it should be removed from the stable and rapidly filtered, aerated, and cooled. During these processes, as well as during any after-operation in the whole process of handling milk two conditions should be fulfilled as nearly as possible: the exclusion of dust and the maintenance of a low temperature. It would seem almost unnecessary to urge the use of clean dairy utensils, and yet, unfortunately, it is necessary so to do. The water with which they are washed is often richly seeded with germs and not infrequently befouled by barnyard sewage. Several epidemics of typhoid fever have been directly traced to the washing of milk-cans in water which was contaminated by typhoid fever discharges. The vessels should be exposed to boiling water or to steam for at least five minutes, or if this is not practicable to water at a lower temperature for as much as twenty minutes.

In no case should the temperature of the water be below 160° F., and the more nearly it approaches the boiling-point the better. This temperature kept up for twenty minutes is certainly fatal to all forms of disease-producing germs, including the tubercle bacillus, except those few which are spore-bearing, such as the anthrax and tetanus bacilli.[1]

[1] THERMAL DEATH-POINT OF SOME OF THE MOST IMPORTANT GERMS.

Sternberg's Manual of Bacteriology.

| | Time of Exposure. |
|---|---|
| Bacillus of diphtheria, 58° C. | 10 minutes. |
| " glanders, 55° C. | 10 " |
| " typhoid fever, 56° C. | 10 " |
| Diplococcus of pneumonia, 52° C. | 10 " |
| Spirillum of Asiatic cholera, 52° C. | 4 " |
| Bacillus coli communis, 60° C. | 10 " |
| " acidi. lactici, 56° C. | 10 " |
| Styphylococcus pyogenes aureus, 58° C. | 10 " |
| " " albus, 62° C. | 10 " |
| Streptococcus pyogenes, 54° C. | 10 " |

Forster: Quoted by Russell.

| | |
|---|---|
| Bacillus of tuberculosis, 65° C. | 30 minutes. |
| " " 68° C. | 15 " |
| " " 75° C. | 10 " |

A study of milk-epidemics reveals another important fact, that in many of them the milk was seeded with the germs of the disease by being handled in the dwelling-house of the dairyman. The obvious lesson from this is that the milk should be handled entirely in a building kept solely for that purpose. When once seeded with bacteria the most important point in the multiplication of the germs is the influence of temperature—a low temperature inhibiting or preventing their growth, while a temperature slightly below that of the body being most favorable to the development of the majority of germs found in milk. One of the great difficulties in obtaining good milk in large cities is the distance between the dairy-farm and the city, so that it is difficult to obtain milk which is not at least twenty-four hours old, and is frequently thirty-six to forty-eight hours old. For this reason, and for the reason that it is practically impossible to maintain milk at a low temperature during so long a time, including the long railroad journey, I advocate strongly pasteurization[1] of milk on the farm. This is considered unnecessary by some, who advocate the utmost care in handling as a substitute, but when it is remembered that in dairies where the utmost precautions are observed under the most careful management, and where the average number of germs does not exceed 3000 to 5000 per cubic centimetre, the number will occasionally reach as high as 200,000 per cubic centimetre, it will be seen that nothing can take the place entirely of pasteurization or sterilization. It should be remembered, however, that sterilization cannot make a bad milk good. It may indeed destroy its infecting power, and to a certain extent the poisonous products formed by bacterial life, but not entirely. Consequently, the sterilization is best done immediately after withdrawing, the milk being put into sealed glass jars preferably, after which it may be kept without injury almost indefinitely. Where pasteurization is practised the good effects are retained only when the milk is rapidly cooled

[1] The terms pasteurization and sterilization are not used as synonymous. Pasteurization is an incomplete or partial sterilization, the degree of heat employed being sufficient to kill all pathogenic germs except those bearing spores, and also the great majority of those which bring about lactic acid fermentation. In practice, as applied to milk, the degree of heat to be used is limited by the production of the "boiled milk" taste, which becomes permanent at about 70° C. (158° F.); while, on the other hand, it must be sufficient to kill the tubercle bacillus, which is the most resistant of the disease-producing germs likely to be found in milk. This bacillus is killed by a temperature of 68.3° C. (155° F.) after an exposure of fifteen minutes; hence, for perfect safety, we heat the milk to 70° C. for twenty minutes. As some spores survive this process, the milk must be cooled, and kept cool subsequently, to prevent their development.

By sterilization is meant the complete destruction of germ-life. This is effected by exposure to the boiling temperature, or even to steam under pressure. Milk so treated will remain sweet indefinitely, even at high temperatures.

and kept cool, and experiment has shown that the temperature should be below 10° C. (50° F.).

Where it is impossible to pasteurize on the farm the milk may be sent in bulk as rapidly as possible to a central station in the city, and there be bottled and pasteurized. The use of the glass jar in the delivery of milk seems to me a great advance over the old system of can-delivery. The constant opening of the large milk-can on the street in all sorts of weather, the atmosphere being often laden with dust made up largely of the droppings of animals on the streets, and containing myriads of fermentative and putrefactive bacteria, offers a constant source of contamination to the milk, which becomes worse and worse at each opening. While there are theoretical dangers in the use of bottles, due to their being carried into the sick-room, and then being imperfectly sterilized before being used again, the experience with them has been most satisfactory, and I have not been able to find a single instance in which disease could be attributed to the use of bottled milk. On this point I am told, by probably the most advanced dairyman in this country, that in an experience extending over twelve years, in which he has made the milk-supply for invalids and for children a specialty, although he sends out from 8000 to 10,000 bottles of milk each day, he has yet to hear of the first instance of the spread of disease from one room to another, or from one nursery to another—certainly very strong evidence in favor of the bottle.

To sum up: milk should be delivered to the consumer in as nearly normal condition as possible. This is attained by the proper care of the cow on the lines laid down, care of the milker as regards his person and clothing, and care of the milk after being drawn. In one word, cleanliness in all things is the secret of a good milk-supply, and if this can be strictly observed such artificial aids as pasteurization are not so necessary. In actual practice, however, I believe that pasteurization will give the best results.

